# Trauma and Public Mental Health: A Focused Review

**DOI:** 10.3389/fpsyt.2019.00451

**Published:** 2019-06-25

**Authors:** Rolf J. Kleber

**Affiliations:** ^1^Utrecht University, Utrecht, Netherlands; ^2^Arq Psychotrauma Expert Group, Diemen, Netherlands

**Keywords:** trauma, posttraumatic stress disorder, resilience, public mental health, war, disasters, violence

## Abstract

Psychological trauma has developed into a very common concept in the scientific community, in mental health care, as well as in popular language and mass media. The purpose of this article is to show the relevance of the discipline of traumatic stress studies to the field of public mental health by examining central concepts and findings concerning trauma and its aftermath and examining implications for public mental health. Attention is paid to the diagnosis of posttraumatic stress disorder (PTSD) and the construct of resilience as well as to specific areas of public mental health activities. A public mental health perspective will help to develop preventive approaches to trauma and extend the impact of various forms of interventions. It will also make clear that trauma care will have to consider the community and the society at large.

## Introduction

Psychological trauma has developed into a very popular concept in the scientific community, in the world of mental health care, as well as in common language and mass media. The consequences of various shocking events—violence, disasters, acts of terrorism, accidents, and war—receive frequent and enduring attention. The number of scientific and clinical publications has increased enormously and in many media programs ample attention is paid to victims and others affected by these events. The purpose of this article is to show the relevance of the discipline of traumatic stress studies to the field of public mental health by examining central concepts and findings concerning trauma and its aftermath and examining implications for public mental health.

## What Is a Traumatic Event?

Traumatic events involve the confrontation with war, violence, disasters, sudden loss, serious illness, and other overwhelming and disturbing events. According to the psychiatric classifications [of the *International Classification of Diseases* of the World Health Organization (ICD-11) and the *Diagnostic and Statistical Manual of Mental Disorders*, fifth edition (DSM-5)], a traumatic event is defined as the exposure to: death, threatened death, actual or threatened serious injury, or actual or threatened sexual violence (1, 2).

Phenomenologically such an event can be characterized by an extreme sense of powerlessness as well as a disruption of beliefs and expectations. The individual has lost control over the situation and is to a large extent a victim of the circumstances and/or other people (i.e. the perpetrator). In “Jenseits des Lustprinzips” (1920), Freud already posed: “the essence of a traumatic situation is an experience of helplessness that is brought about either externally or internally.” At the same time, he or she is confronted with a shattering of basic assumptions. The self-evidence of one’s life is gone. The sense of invulnerability, the idea of the benevolence of the world, and the idea that other people can be trusted are devastated. The obvious certainties of life have disappeared. The images one holds of oneself and the environment no longer adequately fit the new situation. In the words of Janoff-Bulman (3), basic assumptions have been shattered.

Exposure to traumatic events is not rare, as has been consistently found in epidemiological studies. The World Mental Health Surveys of adults were carried out among nearly 70,000 participants from 24 countries ranging in economic status from low to high (4). These data showed that at some time in their life 70.4% of the respondents had experienced at least one type of a traumatic event. The specific rates were: 14% had experienced intimate partner or sexual violence, 34.3% accidents or injuries, 22.9% physical violence, 13.1% war-related events, 34.1% the unexpected or traumatic death of a loved one, and 35.7% experienced traumas that happened to loved ones (e.g., serious illness of a child). As Kessler et al. (4) stated, these findings make clear that it is rather normal to be exposed to a very upsetting event in one’s lifetime.

## Rise and Bloom of the Concept of Post-Traumatic Stress Disorder

The concept of *posttraumatic stress disorder* (PTSD) is nowadays so much used that it dominates most thinking about the consequences of violence, disaster, and being a refugee. That development is quite unique as the attention for trauma was very meager just 40, 50 years ago. In the 1970s, the United States became increasingly confronted with the psychological and social difficulties of the nearly three million veterans who had fought in Vietnam. They suffered from nightmares, depression, relationship problems, et cetera. However, because of the ambivalent perspective on the Vietnam War, authorities and professionals were rather reluctant to acknowledge these difficulties, but the increasing concern led to the introduction of the term post-traumatic stress disorder in the anxiety disorders section of the *Diagnostic and Statistical Manual of Mental Disorders* (DSM-III) (5).

The diagnosis of PTSD is directly linked to experiencing or witnessing a traumatic event such as a natural disaster, a serious accident, a terrorist act, war/combat, rape, or other violent assault (criterion A). According to DSM-5 (1), PTSD consists of four categories of symptoms. Re-experiencing the traumatic event (criterion B) is manifested in symptoms such as intrusive memories, distressing dreams, flashbacks, or distress or physiological reactions upon exposure to cues of the trauma. The other categories are symptoms of avoidance of the reminders of the trauma (criterion C), alterations in memories or mood associated with the trauma (criterion D), and finally clear alterations in physiological arousal and reactivity [criterion E; see further Ref. (1)].

Recently a relatively different definition of PTSD has been introduced in the new version of the International Classification of Diseases of the World Health Organization (2018). It consists of three categories (reexperiencing, avoidance, hyperarousal) with only two core symptoms in each category. The definition may be more flexible to use (allowing for cultural variation and clinical judgment) but is less detailed and comprehensive. Empirical studies have found that prevalence rates may vary between the two classifications and there is often a lack of overlap (i.e., not the same individuals are classified as PTSD patients by the two classifications). This is a major concern for the field of traumatic stress studies. It is a challenge for future research to unravel the differences and to create order (6), as most research is conducted with the DSM definition while most countries in the world use instead ICD.

Prevalence figures for PTSD vary enormously, according to the nature of the events, various risk factors, the time of measurement, and the instruments used. In general, PTSD occurs more when an aggressor is involved (in the case interpersonal violence), for persistent and extended events (e.g., internment, sexual abuse) and for socially charged events (e.g., rape). Among Dutch veterans who were confronted with war violence during deployment in Iraq, the figures of current prevalence varied between 3 and 4% (7). Nine percent of American Vietnam veterans had current PTSD 20 years after the war (8). Figures on PTSD in studies of disasters (man-made, technological, as well as natural) varied mostly around 5% to 15% (9). The prevalence findings also fluctuated strongly in studies of sexual violence, although the rates are generally higher than after other events: between 3.7% and 65%. The already mentioned World Mental Health Surveys (4, 10) determined that intimate partner or sexual violence (such as rape) was a very frequent cause of PTSD. Nevertheless, the unexpected death of loved ones represented the most frequent cause of event-related psychopathology within the general population because of the high frequency with which people experience such a loss. About this last mentioned finding, it is relevant to remark that there is a close relationship between trauma and loss and consequently between PTSD and complicated grief, but they are not similar (11). The concepts of Persistent Complex Bereavement Disorder (1) and Prolonged Grief Disorder (2) as result of the death of a family member or a close friend are included in DSM-5 and ICD-11, respectively.

In a comprehensive and systematic analysis (12) prevalence rates of PTSD and depression were identified from 181 surveys comprising 81,866 refugees and other conflict-affected persons from 40 countries exposed to humanitarian emergencies. Again, rates of reported PTSD and depression showed large inter-survey variability. The prevalence estimates derived from the methodologically most robust surveys provided rates between 13% and 25% for PTSD, as assessed by Steel et al. (12). The risk of PTSD among refugees was increased by experiencing torture and sexual violence, having a higher age, being a woman, and through a long stay in different asylum seekers centers.

Despite the increased knowledge about PTSD and despite the positive results of therapeutic treatments for PTSD, there are various dilemmas and challenges. First, there is a large comorbidity, as most patients also suffer from depression, substance abuse, or other disorders (6). Furthermore, defining the borderline between normal and abnormal behavior after trauma is difficult. Moreover, although this is matter of heavy debate among researchers and clinicians, PTSD is sometimes an overstretched concept in the sense that normal responses to stressful life events are labelled as disorder. Difficulties of diverse groups—from refugees to veterans—are attributed too much or too easily to traumatic events and their resilience is underestimated (13).

## Impact on the Individual

Regarding a public mental health perspective, it is highly relevant to understand that the majority of people exposed to serious live events does not develop disorders. However, this does not mean that they will not suffer from symptoms and difficulties. Most people will experience responses such as intrusions, nightmares, startle reactions, and numbness (14). Findings from large epidemiological studies of disaster victims have made this clear. In 2000 the Netherlands were confronted with a disastrous explosion of a fireworks container area in the middle of a neighborhood. A comprehensive and longitudinal study was conducted among the inhabitants. In the investigation of post-disaster reactions (15) it was found that most inhabitants suffered from various serious symptoms (especially depression, fears, re-experiences, physical symptoms) in the first 2 to 3 weeks after the explosion. At least 87% of the affected residents were highly affected in those first weeks after the disaster.

These responses can be considered functional and normal, as has been made clear in emotion theories (16). People are afraid that it will happen again. They do not feel safe anymore and are constantly alert for danger. They are angry because of the neglect of the responsible authorities or they feel rage in the direction of the perpetrator. They react easily irritated at remarks of other people. They blame themselves for being there at the moment of the disaster or having not done anything to prevent the situation. They feel despaired because of the death of loved ones and the loss of material goods. They have the impression that other people do not understand their distress and sorrow and feel estranged from others. Nevertheless, the intensity and frequency of these distressing and painful responses do not reach the level of disorder.

Although DSM-5 recognizes the possibility of the occurrence of Acute Stress Disorder (ASS) in the first days after an event, a diagnosis that overlaps with PTSD, this diagnosis is rarely used in clinical practice as well as research. Patients with ASS usually report numbness, problems with memory, sleep and concentration, irritability, fears, or anxiety and have frequent re-experiences of the event. The usefulness of acute stress disorder as a classification is controversial in the literature (17). The difference with normal reactions to a major life event and with PTSS, apart from the time criterion (ASS can only be used for disturbances in the first 4 weeks after the experience), is not yet confirmed adequately.

As mentioned above, the world does not make sense any more after such a traumatic experience. Already in the 1940s the psychiatrist and concentration camp survivor Victor Frankl stated that the search for meaning played a crucial role in adaptation to threatening events (18). Cognitive approaches to trauma (e.g., 19) state that successful processing of the traumatic experience takes place when new information (e.g., the implication of the traumatic experience) is assimilated into existing structures or models. Unsuccessful processing occurs when the trauma-related information is not integrated into existing beliefs concerning self-image and world views (20). In low control situations not amenable to direct repair or problem-solving, such as trauma, loss, and serious illness, meaning-making is often the most adaptive strategy. As Schok et al. (20) have argued, meaning can be created by answering the question why an event occurred and why it happened to the person. It can also be operationalized as considering the ways in which one’s life changed because of the event and assessing the extent to which one has “made sense of” the experience. Research findings have indicated that these two processes of finding a cause for the extreme event and finding personal benefits in the traumatic experience play independent roles in adjustment after trauma (21, 22). Therefore, the attempt to find meaning discloses itself twofold: in searching for an answer to the question why it happened as well as in rethinking one’s attitudes and priorities to restructure one’s life along more satisfying lines (23).

## Impact on the Community

Violence, disasters, accidents, and war are also stressful events for the community. Consequently, the impact of traumatic events goes beyond those who are directly exposed to the event and affects close relationships, the social environment, and the society at large. A human being does not live in a vacuum. He or she is surrounded by others. And those others will be confronted with the traumatic event and its aftermath too. This holds true for an event that struck an individual, such as a rape. Others hear about the event, perceive the suffering of the victims, and must cope with the implications. Naturally, it holds also true for events that struck a large group of people. For instance, a disaster undermines the social fabric of a community. It can lead to dissolution of social networks and to forced or voluntary migration. Regarding the health care system, it can lead to a disruption of the provision of social services and an erosion of the health care infrastructure (24, 25).

On the other hand, the social environment can stimulate recovery after trauma. The perception of social support has been found to be an influential factor for the effects of traumatic events on the individual as well as the community. For instance, studies undertaken after disasters have shown that social support had a significant stress-buffering effect for post-traumatic problems. Furthermore, a comprehensive meta-analysis (26) has shown that lack of social support systems and lack of sharing of emotions are significant risk factors for mental health disturbances.

## The Other Side of Trauma: Resilience

The finding that most people confronted with extreme life events did not develop disturbances like PTSD created interest in the phenomenon of *resilience*. This concept has been widely used in recent years in the scientific and clinical world. Resilience refers to a dynamic process involving positive adaptation to one’s circumstances in the face of significant adversity, as defined by Luthar and Cicchetti (27). However, there are various understandings of resilience [see Ref. (28)]. Resilience may be treated as a quality, a personal trait, a process, and an outcome. While, for example, some researchers conceive of resilience as a multiply determined developmental process that is not fixed, others use measures of trait resilience, which favors the assumption that resilience is a personality attribute [see for the different views Ref. (29)].

Investigation of resilience can lead to useful avenues for intervention. The concept offers a different perspective on risk and protection. Focusing on what makes individuals strong rather that what makes them weak may aid to understand what helps them to maintain their mental health. Sleijpen and colleagues examined strategies of young refugees in dealing with negative experiences (28). Their findings revealed that young refugees living in the Netherlands were affected by memories of traumatic events experienced in their country of origin or during the flight, but that current stressors, especially for young people without a residence permit, played a more significant role in determining their psychological well-being. The participants in this study used the following four strategies to deal with traumatic experiences and current stressors: (1) acting autonomously, (2) performing at school, (3) perceiving support from peers and parents, and (4) participating in the new society. These strategies helped the young refugees to strengthen their sense of power and control, they gave them some distraction, and they supported or sustained their spirit within the family unit and the new society [see Ref. (28)].

## Prolonged Aftermath

For public mental health initiatives, it is important to realize on the one hand the significance of the resilience of people affected, but at the other hand also the fact that disturbances can last for a very long period. Difficulties do not always disappear in time. Sometimes they may last for a very long time. 10 years after the Enschede Fireworks disaster still 6.7% of a representative sample of the inhabitants of the neighborhood had an indication of chronic disaster-related PTSD (30). 40 years after the Vietnam War (31), prevalence rates of PTSD were 4.5% (male USA veterans) and 6.1% (female veterans). In the World Mental Health Surveys (4), it was also found that PTSD symptoms typically were quite persistent.

The long-term aftermath of trauma can be illustrated with the following research finding. A large community-based sample of child survivors from World War II was compared with a reference group from the Dutch population as well as with clinical groups (32). These children survived internment in the Japanese camps in the former colony of the Dutch East Indies (now Indonesia) during the period 1942–1945 (and afterwards). Most of them were forced to migrate to the Netherlands in the 1940s and 1950s. Long-term sequelae of the persecution were studied by standardized questionnaires on posttraumatic responses, general health, and dissociation. Compared with control individuals of the same age that lived through the German occupation in the Netherlands during World War II, the child survivors from the former Dutch Indies reported significantly more traumatic experiences and mental health disturbances approximately 50 years after the war. 23 percent of these now adult child survivors in the community sample had an indication of PTSD related to the events in World War II [see Ref. (32)].

These results underline the long-term aftermath of traumatic experiences. This conclusion is also relevant for the many war refugees who migrated to Europe in recent years. In the field of psychotraumatology, there has been a continuing debate about the extent to which diagnostic criteria for PTSD adequately cover the posttraumatic symptomatology experienced by individuals exposed to prolonged, repeated, and interpersonal traumatic events, such as occurring in situations of domestic violence, war, and torture (33). This symptomatology is more complex, more severe, and more invasive than that captured by the classic PTSD diagnosis. This manifestation of psychopathology is referred to as *complex PTSD* (CPTSD). In the 11th version of the ICD (2), this concept is added as a formal diagnosis comorbid to PTSD. It consists of impairments in three domains: difficulties in emotion regulation, negative beliefs about oneself, and difficulties in sustaining relationships. The concept is attractive as it focuses on personal changes due to the confrontation with enduring violence and oppression, but it is as such also rather (too) close to personality disorders while it is not clear whether the addition of this new diagnostic concept is really required beyond PTSD. Research is also still indecisive about these matters. Because of these reasons, a concept such as complex PTSD was not included in DSM-5.

## Public Health Strategies

Public health strategies are aimed at preventing or diminishing mental health problems and addressing the causes of these conditions (see also 10). These strategies are active on multiple levels: individual, family, community, and society. Unfortunately, public health care of trauma is a rather underdeveloped area (see Magruder et al., 2107), especially in contrast to the field of treatment of trauma-related disorders, in particular PTSD. Psychotherapies of PTSD have been found successful, as has been shown by many RCT’s (randomized controlled trials) and meta-analyses, in comparison with control groups and placebo treatments [e.g., Ref. (34)]. Most evidence has been found for trauma-focused cognitive behavioral therapy and Eye Movement Desensitization and Reprocessing (EMDR), and to a somewhat lesser extent Narrative Exposure Therapy and Brief Eclectic Psychotherapy for PTSD [e.g., Ref. (35)]. However, here we focus only on public health strategies relevant to trauma care. They are all explicitly aimed at preventing the emergence of health problems or preventing the aggravation of these difficulties.

*Preventing adversity*. First, although it is a truism, one should bear in mind that stopping or avoiding exposure to events that can be experienced as traumatic is a sensible public health strategy. If disasters, accidents, or wars can be prevented by concrete measures, the chance on traumatic experiences and on their negative consequences is, by definition, taken away.

*Creating awareness and recognition*. Secondly, creating awareness and acknowledgment concerning the impact of trauma on the population is a strategy by which difficulties can be prevented. Psycho-education with the help of brochures is an obvious example of this. Similarly, so-called silent journeys by a community after a violent crime in their neighborhood and creating monuments (“lieux de memoire”) are just as exemplary regarding recognition and appreciation for the people affected by trauma.

The increase of awareness through public health campaigns has been suggested for combatting child sexual abuse (36). Public health campaigns serve to help the identification of affected children and to facilitate the recognition of their difficulties by adults. Such services imply the awareness of the impact of traumatic experiences in diverse domains (individual, societal), the recognition of signs and symptoms, the integration of knowledge about trauma in public health programs, and the prevention of re-traumatization for their users. In this respect, technology-based interventions (e.g., online platforms, social media, mobile applications) can be advantageous. Such campaigns of awareness and recognition should be accompanied by mental health services with adequate interventions for abused persons looking for care. Nevertheless, despite their positive aims and effects, at the same time, all these campaigns may have a downside: they can lead to complications, such as the risks of promoting an unnecessary victim status and medicalizing complaints of the affected persons, resulting in a reduction of the potential for spontaneous recovery (36, 37).

*Strengthening resilience*. The third group of strategies is focused on bolstering resilience and stimulating self-efficacy. For example, training programs have been developed in the armies of the USA and several European countries to allow military soldiers to deal with the stress of war and to be more resistant to the intense and overwhelming events they will be confronted with.

*Counseling*. Fourth, counseling people confronted with traumatic events is a well-known intervention in the field of trauma care. Various forms of secondary preventive interventions providing practical care, support, and information have been designed for employees of organizations confronted with extreme events in the work setting, such as the police and banks, but also for victims of large-scale acts of terrorism. These interventions consist of a couple of protocolled sessions in the first 2 or 3 months after a calamity [see for an overview Ref. (38)]. They are considered as quite helpful, although controlled research is mostly lacking. This intervention should not be confused with so-called psychological debriefing, a typically single session of group counseling directly after a disaster or an act of violence. If this form of support is focused too much on the ventilation of emotions, debriefing has been found to have a negative impact: posttraumatic reactions and depressive feelings are worsened and very early exposure to the trauma material may interfere with natural recovery processes (9, 38).

*Reconciliation*. A related form of post-trauma care are large-scale programs to reconcile people after large-scale conflicts. For instance, a civil war divides families, communities, and nations, often pitting one neighbor against another. Distrust, resentment, and anger dominate post-war society, just as much as passivity and emotional numbness. That is why programs are designed to reconcile people (perpetrators and victims) and to restore trust, connectedness, and social cohesion.

Such truth and reconciliation efforts were conducted in Sierra Leone (39). Community-level forums in 200 villages were set up in which victims detailed war atrocities and perpetrators confessed to war crimes. Research using a randomized control trial among more than 2,000 individuals showed that reconciliation led to greater forgiveness of perpetrators and strengthened social capital: social networks were larger, and people contributed more to public goods in treated villages. However, there were also negative psychological costs next to the positive societal benefits. The reconciliation intervention also worsened psychological health, increasing depression, anxiety, and PTSD in the same villages. For a subset of villages, outcomes were measured 9 months and 31 months after the intervention. Results showed that both positive and negative effects persisted into the longer time horizon. These findings suggest that policy-makers should be careful with reconciliation processes and must find ways to restrict the emphasis on emotions.

*Policy making*. Finally, there is the overarching field of policy making, a rather underdeveloped topic in trauma care. As Magruder et al. (40) stated, public policies should be formulated to prevent traumatic events, to understand risk and protective factors, to provide early intervention services for individuals and communities at risk of post-trauma maladjustment, and to shape societal norms to eliminate stigma. Extra priorities for improving mental health include a focus on adequately training researchers and professionals, supporting international collaborations, and encouraging scientists to share their expertise with policymakers. Furthermore, integration of physical and mental health care is especially important, as trauma-exposed individuals often seek help in primary care rather than mental health settings. Consequently, posttraumatic disturbances may go undiagnosed.

## Finally

A public mental health perspective will help to develop preventive approaches to trauma and extend the impact of various forms of interventions. It will also make clear that trauma care will have to consider the community and the society at large. The concept of trauma is an attractive concept. It refers to both spectacular and shocking events that receive huge attention, such as acts of terrorism and large-scale calamities. Something dramatic happens that could happen to anyone: the cause appears to be clear and the responsibility appears to lay elsewhere. One could argue that traumatic experiences show us the limits of our abilities to master our lives and that they defy our efforts to control the circumstances. However, the concept of trauma is also a dangerous concept. It is often used too easy and too quick. Not every stressful event is a traumatic experience and not every person confronted with war, disaster, or terror is traumatized. Overstretching the concept may create the risk of medicalization of regular difficulties of the afflicted people and ignoring the self-reliance and the adaptive skills of them.

## Author Contributions

I am the sole author. I designed and wrote the manuscript.

## Conflict of Interest Statement

The author declares that the research was conducted in the absence of any commercial or financial relationships that could be construed as a potential conflict of interest.
